# The prognostic significance of tumour cell detection in the peripheral blood versus the bone marrow in 733 early-stage breast cancer patients

**DOI:** 10.1186/bcr2898

**Published:** 2011-06-14

**Authors:** Timothy J Molloy, Astrid J Bosma, Lars O Baumbusch, Marit Synnestvedt, Elin Borgen, Hege Giercksky Russnes, Ellen Schlichting, Laura J van't Veer, Bjørn Naume

**Affiliations:** 1 Division of Experimental Therapy, The Netherlands Cancer Institute, Plesmanlaan 121, Amsterdam, 1066 CX, The Netherlands; 2 Department of Genetics, Institute for Cancer Research, Oslo University Hospital Radiumhospitalet, Postboks 4953, Nydalen, 0424 Oslo, Norway; 3 Biomedical Research Group, Department of Informatics, University of Oslo, Postboks 4953, Nydalen, 0424 Oslo, Norway; 4 Division of Surgery and Cancer Medicine, Oslo University Hospital Radiumhospitalet, Postboks 4953, Nydalen, 0424 Oslo, Norway; 5 Department of Pathology, Oslo University Hospital Radiumhospitalet, Postboks 4953, Nydalen, 0424 Oslo, Norway; 6 Institute of Clinical Medicine, University of Oslo, Postboks 4953, Nydalen, 0424 Oslo, Norway

## Abstract

**Introduction:**

The detection of circulating tumour cells (CTCs) in the peripheral blood and disseminated tumour cells (DTCs) in the bone marrow are promising prognostic tools for risk stratification in early breast cancer. There is, however, a need for further validation of these techniques in larger patient cohorts with adequate follow-up periods.

**Methods:**

We assayed CTCs and DTCs at primary surgery in 733 stage I or II breast cancer patients with a median follow-up time of 7.6 years. CTCs were detected in samples of peripheral blood mononuclear cells previously stored in liquid-nitrogen using a previously-developed multi-marker quantitative PCR (QPCR)-based assay. DTCs were detected in bone marrow samples by immunocytochemical analysis using anti-cytokeratin antibodies.

**Results:**

CTCs were detected in 7.9% of patients, while DTCs were found in 11.7%. Both CTC and DTC positivity predicted poor metastasis-free survival (MFS) and breast cancer-specific survival (BCSS); MFS hazard ratio (HR) = 2.4 (*P *< 0.001)/1.9 (*P *= 0.006), and BCSS HR = 2.5 (*P *< 0.001)/2.3 (*P *= 0.01), for CTC/DTC status, respectively). Multivariate analyses demonstrated that CTC status was an independent prognostic variable for both MFS and BCSS. CTC status also identified a subset of patients with significantly poorer outcome among low-risk node negative patients that did not receive adjuvant systemic therapy (MFS HR 2.3 (*P *= 0.039), BCSS HR 2.9 (*P *= 0.017)). Using both tests provided increased prognostic information and indicated different relevance within biologically dissimilar breast cancer subtypes.

**Conclusions:**

These results support the use of CTC analysis in early breast cancer to generate clinically useful prognostic information.

## Introduction

In recent years breast cancer survival rates have been steadily increasing, partly due to earlier diagnoses as a result of increased awareness and widespread mammography screening programmes. Despite these advances, approximately one-third of patients will develop distant metastasis, which represents the terminal step in the progression of the disease. The relative paucity of accurate prognostic tests has made it difficult to identify these high-risk patients to allow for more optimized adjuvant treatment decisions. Similarly, many patients at low risk of developing disseminated disease undergo toxic adjuvant chemotherapy treatments that are of little benefit. There is consequently a need for new prognostic tests with significantly increased sensitivity and specificity, particularly for those patients at the early stages of their disease.

Tumour cells in the bone marrow (disseminated tumour cells (DTCs)) or circulating in the peripheral blood (circulating tumour cells (CTCs)), are potential progenitors of distant metastasis and may, therefore, represent important targets of such tests [1-12]. Indeed, the detection of CTCs and DTCs has been associated with both disease progression in metastatic breast cancer [13-15], as well as disease recurrence and distant spread in early-stage breast cancer [1,3,5,6,16]. Bone marrow has traditionally been the primary compartment in which the prognostic value of the detection of these cells has been investigated, as it is a common homing organ for tumour cells of epithelial origin [17]. While the detection of DTC is of prognostic significance [1,3,5], it has the inherent disadvantage of needing an invasive procedure for sample collection and, additionally, is less suitable for serial sampling for monitoring adjuvant treatment response. The use of peripheral blood as an alternative sampling material has, therefore, gained significant interest in recent years as it does not suffer from these drawbacks. However, there remains a need for good quality studies to confirm the clinical relevance of CTCs in larger patient cohorts, particularly in comparison to DTCs. So far relatively few such studies in early breast cancer have included mature outcome data. Furthermore, only limited data exist on the comparison of CTCs and DTCs in this context, and studies have shown variable results [7,18,19]. Whether CTC detection could replace DTC detection is still an open question, and the value of CTCs versus DTCs may differ among patients as a consequence of different tumour biology and/or dissemination characteristics.

In the current study, we used our previously-developed [2,20] multi-marker QPCR-based CTC assay on thawed samples of liquid nitrogen-stored peripheral blood mononuclear cells (PBMC) from 733 early-stage breast cancer patients collected at the time of diagnosis. In addition, this patient series previously had bone marrow samples assayed for DTCs [1], which afforded an opportunity to directly compare the prognostic relevance of CTC and DTC detection in the same large patient group.

## Materials and methods

### Patient and sample selection

Between May 1995 and December 1998, samples of peripheral blood and bone marrow were collected from 920 stage I-II primary breast cancer patients immediately prior to primary surgery, at five hospitals in Norway (Ullevål University Hospital (now Oslo University Hospital Ullevål), Norwegian Radium Hospital (now Oslo University Hospital Norwegian Radium Hospital), Bærum Hospital, Aker University Hospital, and Buskerud Hospital), as described previously [1]. After excluding patients from whom peripheral blood mononuclear cells (PBMC) were either unavailable for this study (*n *= 149) or who were eligible for and/or received preoperative systemic therapy (pT3/4 patients; *n *= 38), which would likely distort the true CTC levels, PBMC were thawed from liquid nitrogen storage for CTC analysis. Bone marrow samples from the same patients (*n *= 733) had previously been analysed for the presence of DTCs [1]. Patients were treated with breast-conserving surgery or breast ablation and axillary clearance in addition to adjuvant therapy where required, as described in Table 1. Systemic adjuvant treatment was given according to the national guidelines between 1995 and 1998. Patients with node negative disease having either tumours ≤ 2 cm or grade 1 tumours 2 to 5 cm in diameter did not receive systemic adjuvant treatment. In addition, patients > 65 years of age did not receive chemotherapy. The standard adjuvant chemotherapy regimen consisted of six cycles (3 qw) of intravenous cyclophosphamide 600 mg/m^2^, methotrexate 40 mg/m^2^, and fluorouracil 600 mg/m^2^. Ten patients received high-dose chemotherapy. Tamoxifen was used as endocrine therapy in hormone receptor-positive patients. Radiotherapy was offered after breast-conserving surgery for all patients and after mastectomy for node-positive premenopausal patients and postmenopausal patients with four or more positive lymph nodes. The follow-up consisted of clinical examination at 6- to 12-month intervals at the hospital outpatient departments or by the patients' primary doctors, with mammography annually. Further diagnostic work-up was performed only if the patients had symptoms or signs of progression. The median follow-up time for metastasis-free survival was 7.1 years, and for breast cancer-specific survival 8.3 years. Written informed consent was obtained from all participants and the study was approved by the Regional Ethic Committee.

**Table 1:** Clinical characteristics of the stage I to II breast cancer patients included in the study

		All patients		CTC positive		CTC negative		
		Number	Percent	Number	Percent	Number	Percent	*P*-value*
*Number:*		733	100%	58	7.9%	675	92.1%	
*Age:*	< 35	15	2.0%	1	1.7%	14	2.1%	0.503
	35 to 45	89	12.1%	10	17.2%	79	11.7%	
	45 to 55	203	27.7%	18	31.0%	185	27.4%	
	> 55	426	58.1%	29	50.0%	397	58.8%	
*Tumour size:*	pT1 (< 2.0 cm)	405	55.3%	30	51.7%	375	55.6%	0.373^§^
	pT2 (2.0 to 5.0 cm)	225	30.7%	20	34.5%	205	30.4%	
	pT3 (> 5.0 cm)	2	0.3%	2	3.4%	0	0%	
	pTX	60	8.2%	3	5.2%	57	8.4%	
*Histological grade:*	Grade 1	187	25.5%	9	15.5%	178	26.4%	0.186
	Grade 2	350	47.7%	29	50.0%	321	47.6%	
	Grade 3	186	25.4%	18	31.0%	168	24.9%	
	Unknown	10	1.4%	2	3.4%	8	1.2%	
*ER status:*	Positive	516	70.4%	31	53.4%	485	71.9%	**0.002**
	Negative	179	24.4%	24	41.4%	155	23.0%	
	Unknown	38	5.2%	3	5.2%	35	5.2%	
*PR status:*	Positive	401	54.7%	25	43.1%	376	55.7%	**0.046**
	Negative	288	39.3%	30	51.7%	258	38.2%	
	Unknown	44	6.0%	3	5.2%	41	6.1%	
*HER2 status:*	Positive	83	11.3%	15	25.9%	68	10.1%	**< 0.001**
	Negative	610	83.2%	40	69.0%	570	84.4%	
	Unknown	40	5.5%	3	5.2%	37	5.5%	
*Node status:*	pN0	483	65.9%	34	58.6%	449	66.5%	0.089
	pN1	150	20.5%	11	19.0%	139	20.6%	
	pN2	53	7.2%	9	15.5%	44	6.5%	
	pN3	22	3.0%	3	5.2%	19	2.8%	
	pNX	25	3.4%	1	1.7%	24	3.6%	
*Vascular invasion:*	Yes	116	15.8%	12	20.7%	104	15.4%	0.171
	No	524	71.5%	35	60.3%	489	72.4%	
	Unknown	93	12.7%	11	19.0%	82	12.1%	
*Bone marrow status:*	Positive	86	11.7%	17	29.3%	69	10.2%	**< 0.001**
	Negative	605	82.5%	35	60.3%	570	84.4%	
	Unknown	42	5.7%	6	10.3%	36	5.3%	
*Adjuvant systemic treatment:*	No treatment	391	53.3%	28	48.3%	363	53.8%	0.392
	Chemo +/- Hormonal	155	21.1%	16	27.6%	139	20.6%	
	Hormonal therapy Only	164	22.4%	11	19.0%	153	22.7%	
	Unknown	23	3.1%	3	5.2%	20	3.0%	
*Metastasis:*	Number	149	20.3%	22	37.9%	127	18.8%	**0.001**
	Median time to event	36.4 months		30.8 months		37.2 months		
	Median followup time	84.3 months		78.6 months		85.2 months		
*Breast cancer-specific deaths:*	Number	122	16.6%	19	32.8%	103	15.3%	**< 0.001**
	Median time to event	42.9 months		54.3 months		42.0 months		
	Median followup time	99.2 months		94.1 months		99.2 months		

*P-value (Pearson Chi-squared test).

§ = Chi-squared analysis included pT1 and pT2 tumours only.

CTC, circulating tumour cell; ER, estrogen receptor; HER2, human epidermal growth factor receptor 2; PR, progesterone receptor

### Control subjects

For the CTC study, 16 patients with metastatic breast cancer (M_1 _disease, according to the Union Internationale Contre le Cancer criteria) were included as clinical "positive controls" as based on our previous studies [2] the majority (> 90%) were expected to have circulating tumour cells. They were invited to participate if they were between treatments or soon to start subsequent palliative treatment modality. A total of 28 samples of peripheral blood from healthy controls into which different numbers (5 to 1,000) of cultured breast tumour cells derived from a mixture of six breast cancer cell lines (MCF7, CAMA1, T47D, SKBR3, ZR-75-1, and MDA-MB-231) were also tested in parallel as a second positive control group. Twenty-five healthy (negative) control subjects matched for gender and age and randomly selected from hospital and scientific staff were also freshly collected. Mononuclear cells from these control groups were separated and stored frozen for up to one week to mimic the processing and storage conditions of the patient samples, before being thawed and assayed. For the DTC study, bone marrows from 98 healthy controls were assayed.

### Sampling and processing of peripheral blood (PB)

A total of 50 mL of blood was collected immediately prior to primary surgery. Mononuclear cells, including any tumour cells present, were separated from the whole blood samples using Ficoll Hypaque (BD, Breda, Netherlands) and stored frozen in aliquots of 20 × 10^6 ^cells (except from 30 patients, from which less than 20 × 10^6 ^cells were available) in liquid nitrogen until tumour cell enrichment (10 to 15 years later). Tumour cells were separated from a single aliquot of peripheral blood mononuclear cells (PBMCs) using anti-EpCAM (clone HEA-125) and anti-ErbB2 Micro Beads (MACS^®^; Miltenyi Biotec, Leiden, Netherlands) according to the manufacturer's instructions. In brief, beads were incubated with the PBMCs for 30 minutes at 4°C, after which labelled cells were collected on a magnetic separation column. After removal of the column from the magnetic field, the retained EpCAM^+ ^and/or ErbB2^+ ^cells were eluted, and stored at -70°C in lysis buffer (5 M Guanidine thiocyanate (Merck, Darmstadt, Germany), pH 6.8, 0.05 M tris, 0.02 M EDTA, 1.3% Triton) until mRNA isolation and cDNA synthesis.

### mRNA isolation and cDNA synthesis

Total RNA was precipitated from the cell lysate and dissolved in lysis buffer from the μMACS™ One-step cDNA kit (Miltenyi Biotec). Oligo(dT) Micro Beads were added and the mixture placed onto the μMACS column in the thermo MACS™ Separator (Miltenyi Biotec, Leiden, Netherlands). The isolated mRNA was directly converted into cDNA as per the manufacturer's instructions, with an additional elution with 20 μl of elution buffer, resulting in a total volume of 70 μl.

### Quantitative real-time PCR

Quantitative real-time PCR primers (Sigma Genosys, Cambridge, UK) and 5'-fluorescently FAM-labelled probes (Applied Biosystems, Nieuwerkerk a/d IJssel, The Netherlands) were designed using the Perkin Elmer Primer Express^® ^software (PE, Foster City, CA, USA) based on the published sequences of CK19, p1B, EGP-2, PS2, mammaglobin and SBEM as previously described [20] (Table 2). All primers were designed to be intron-spanning to preclude amplification of genomic DNA. Commercially available primers and probes for the "housekeeping" genes β-actin and glyceraldehyde-3-phosphate dehydrogenase (GAPDH) (Applied Biosystems) were also used.

**Table 2:** Primer sequences for the six marker genes used for CTC detection

Gene	Accession	Sequence	Probe (5'FAM-3'TAMRA)
p1B	[Genbank: L15203]	Sense: CTGAGGAGTACGTGGGCCTG Antisense: AGTCCACCCTGTCCTTGGC	CTGCAAACCAGTGTGCCGTGCC
PS2	[Genbank:X00474]	Sense: GAGGCCCAGACAGAGACGTG Antisense: CCCTGCAGAAGTGTCTAAAATTCA	CTGCTGTTTCGACGACACCGTTCG
CK19	[Genbank:NM002276]	Sense: CTACAGCCACTACTACACGAC Antisense: CAGAGCCTGTTCCGTCTCAAA	CACCATTGAGAACTCCAGGATTGTCCTGC
EGP2	[Genbank:M32306]	Sense: CAGTTGGTGCACAAAATACTGTCA Antisense: CCATTCATTTCTGCCTTCATCA	TTGCTCAAAGCTGGCTGCCAAATGTT
SBEM	[Genbank:AF414087]	Sense: CTCTTGGGGAGTTTTCCATCTTTCTG Antisense: CTTCATCATCAGCAGGACCAGTAG	CCCAGAATCCGACAACAGCTGCTCC
MmGl	[Genbank:AF015224]	Sense: TTCTTAACCAAACGGATGAAACTCT Antisense: GGTCTTGCAGAAAGTTAAAATAAATCAC	TGCTGTCATATATTAATTGCATAAACACCTCAACATTG

Serially diluted cDNA synthesized from the amplified RNA of a pool of 82 snap frozen breast cancer tissues was used to generate standard curves for control and marker gene expression. For all cDNA dilutions, fluorescence was detected from 0 to 50 PCR cycles for the control and marker genes in single-plex reactions, which allowed the deduction of the C_T_-value (threshold cycle) for each product. The C_T_-value is the PCR cycle at which a significant increase in fluorescence is detected due to the exponential accumulation of PCR products and is represented in arbitrary units (TaqMan Universal PCR Master Mix Protocol, Applied Biosystems) [21]. The "housekeeping" genes β-actin and GAPDH were used to confirm reaction efficiency. Each measurement was performed in triplicate. Quality control measures for the PCR reactions included the addition of a genomic DNA control and a non-template control.

### Quadratic discriminant analysis (QDA) 'CTC score' calculation

QDA was used to calculate a 'CTC score' representing CTC presence or absence for each sample, as previously described [22,23], using the expression data of the four best marker genes (CK19, p1B, EGP and MmGl). QDA is a statistical approach to find the combination of quadratic and linear functions of variables (in this case marker genes), which leads to the optimal separation between groups (in this case metastatic breast cancer patients and healthy volunteers). It is a generalization of Fisher's Linear Discrimination Analysis (LDA), which allows only linear functions [24]. A positive discriminant score indicates the presence of tumour cells in a sample, and conversely a negative discriminant score indicates their absence. QPCR measurement and QDA data analysis in this way offer a simple and objective estimate of tumour cell presence in a given sample.

### Sampling and processing of bone marrow (BM)

The processing and analysis of DTC has been previously described [1]. Briefly, BM was collected from patients under general anaesthesia just before primary surgery for suspected breast cancer. A total of 40 mL of BM was aspirated from anterior and posterior iliac crests bilaterally (10 mL per site) and after separation by density centrifugation, mononuclear cells (MNC) were collected and cytospins were prepared (5 × 10^5 ^MNC/slide). Four slides were incubated with the anticytokeratin monoclonal antibodies (mAbs) AE1 and AE3 (Sanbio, Uden, The Netherlands), and the same number of slides were incubated with an isotype-specific irrelevant control mAb. The visualization step included the alkaline phosphatase/anti-alkaline phosphatase reaction, and the slides were counterstained with hematoxylin to visualize nuclear morphology. The cytospins were manually screened by light microscopy by a single pathologist (EB). The determination of the presence of DTC was based on Consensus morphology criteria [25]. In the DTC-positive patients, a median of 1 cell was detected, with a mean of 98 cells (range 1 to 7,500). Of the bone marrow samples from 98 healthy controls, 4 contained ≥ 1 cell (3 with 1 cell, 1 with 2 cells) which were scored positive by the described technique. Those patients with a DTC positive event in the negative control (isotype specific), were excluded from DTC interpretation in the specific test (AE1AE3), which improved the specificity of the DTC analysis.

### Statistical analysis

The primary endpoints for the survival analysis was breast cancer-specific survival (BCSS), which was measured from the date of surgery to breast cancer-related death or otherwise censored at the time of the last follow-up visit or at non cancer-related death, and metastasis-free survival (MFS), measured in the same way. Kaplan-Meier survival curves for time to distant recurrences and breast cancer-specific death were plotted. *P-*values were computed by the log-rank test. Cox proportional hazards regression was used for univariate and multivariate (stepwise backward elimination) analysis of prognostic impact of relevant variables. For statistical analysis, the SPSS (Version 15.0.1; SPSS, Inc, Chicago, IL, USA) software was used.

### Results

#### Detection of CTC

Results from a total of 733 stage I to II breast cancer patients with biomaterial available at the time of surgery for both CTC and DTC analysis are presented. For CTC detection, samples of peripheral blood from a group of healthy individuals (negative controls) and metastatic breast cancer patients (positive controls) were processed the same way as the patient samples. These were used to calculate weights for each of the tumour marker genes used in the CTC assay and to set the threshold of CTC positivity. In an analogous fashion, 28 blood samples from healthy volunteers into which cultured breast tumour cells were spiked were also investigated as positive controls, since all should contain detectable levels of tumour marker gene expression. This sample set generated comparable tumour marker weights to the metastatic patients. This analysis also demonstrated that the assay gave a positive CTC score when as few as one tumour cell per mL was present in the sample (data not shown).

Using the above data to tune the QDA CTC score calculation, 58 of the 733 (7.9%) patients had a positive CTC score and determined CTC-positive, versus 0 out of 25 healthy controls in the negative control group. The CTC result was compared to clinical and histopathological parameters, as well as the DTC status, as described in Table 1. Patients determined to be CTC-positive were significantly more likely to have ER-negative (*P *= 0.002 (Pearson Chi-square test)), PR-negative (*P *= 0.046), HER2-positive (*P *< 0.001) tumours, and have tumour cells present in the bone marrow (*P *< 0.001).

### Circulating tumour cell status and patient outcome

**Figure 1:**
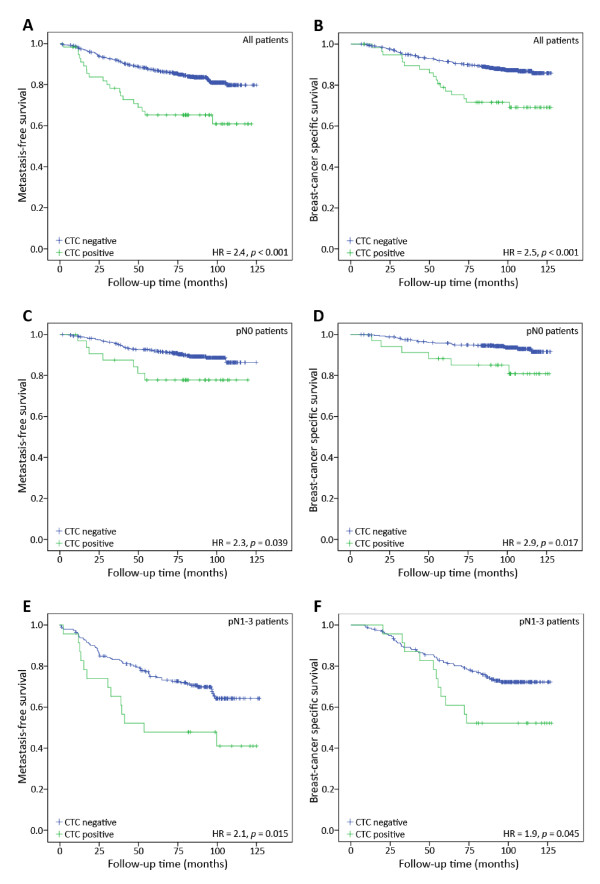
**Metastasis-free survival (**A, C, E**) and breast-cancer specific survival (**B, D, F**) for all,(A, B), lymph-node negative, (C, D), and lymph-node positive (E, F) patients**.

The median observation period for the patients following blood collection was 7.1 years for metastasis-free survival, and 8.3 years for breast cancer-specific survival. During these periods, 149 patients (20.3%) experienced metastasis - 22 (37.9%) of the CTC-positive patients, versus 127 (18.8%) of the CTC-negative patients (*P *= 0.001 (Pearson Chi-square test); Table 1). Cox regression analysis demonstrated that CTC status was a significant predictor of metastasis-free survival (MFS; hazard ratio (HR) = 2.4, *P *< 0.001; 95% confidence interval (CI) = 1.5 to 3.9; Figure 1A; Table 3). Similarly, 122 patients (16.6%) died from their disease during the follow-up period: 19 (32.8%) CTC-positive patients, versus 103 (15.3%) CTC-negative patients. CTC status was, therefore, also a significant prognostic factor for breast-cancer specific survival (BCSS; HR = 2.5, *P *< 0.001; 95% CI = 1.5 to 4.3); Figure 1B; Table 3).

**Table 3:** Univariate Cox regression analysis of CTC and DTC status for metastasis-free and breast cancer-specific survival

	Metastasis-free survival			Breast cancer-specific survival		
Clinical variable	*HR*	*P-value*	*95% CI*	*HR*	*P-value*	*95% CI*
CTC status	2.4	< 0.001	1.5 to 3.9	2.5	< 0.001	1.5 to 4.3
DTC status	1.9	0.006	1.2 to 2.9	2.3	0.001	1.4 to 3.7

CI, confidence interval; CTC, circulating tumour cell; DTC, disseminated tumour cell; HR, hazard ratio

CTC status was next tested in a multivariate Cox regression model, including DTC status and standard prognostic clinical variables (lymph node status, histological grade, tumour size, hormone receptor status, HER2 status, and vascular invasion). In the final model, CTC status remained a significant prognostic factor for both relapse-free survival and breast cancer-specific survival (Table 4).

**Table 4:** Multivariate Cox regression analysis of CTC and DTC status for metastasis-free and breast cancer-specific survival

	Relapse-free survival			Breast cancer-specific survival		
Clinical variable	*HR*	*P-value*	*95% CI*	*HR*	*P-value*	*95% CI*
CTC status (†)	1.8	0.043	1.0 to 3.3	1.9	0.032	1.1 to 3.6
DTC status (†)	*	*	*	*	*	*
Lymph nodes (vs pN0)		< 0.001			< 0.001	
- pN1	2.2	0.001	1.4 to 3.5	2.6	0.001	1.5 to 4.5
- pN2/3	3.2	< 0.001	1.8 to 5.5	3.8	< 0.001	2.1 to 7.0
Histological grade (vs I)		0.053			0.049	
- grade II	2.0	0.094	0.9 to 4.5	1.6	0.324	0.6 to 4.3
- grade III	2.8	0.019	1.2 to 6.8	2.8	0.044	1.0 to 7.7
Tumour size (< 20 mm vs > 20 mm)	1.9	0.002	1.3 to 2.9	2.4	< 0.001	1.5 to 3.8
ER/PR status (‡)	1.7	0.028	1.1 to 2.7	1.9	0.018	1.1 to 3.1
HER2 status (†)	*	*	*	1.8	0.024	1.1 to 3.1
Vascular Invasion (†)	1.6	0.048	1.0 to 2.5	1.8	0.015	1.1 to 3.0

*, not significant in final multivariate model; †, positive versus negative; ‡, both negative versus either or both positive

CI, confidence interval; CTC, circulating tumour cell; DTC, disseminated tumour cell; ER, estrogen receptor; HER2, human epidermal growth factor receptor 2; HR, hazard ratio; PR, progesterone receptor

**Figure 2:**
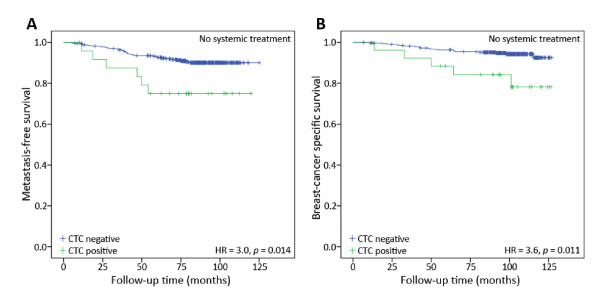
**Kaplan-Meier plot of metastasis-free survival (**A**) and breast cancer specific survival (**B**) in patients who did not receive systemic adjuvant treatment (*n *= 367)**. The CTC-positive patients had a poorer MFS and BCSS than CTC-negative patients (MFS HR = 3.0, 95% CI = 1.2 to 7.1, *P *= 0.014; BCSS HR = 3.6, 95% CI = 1.3 to 9.6, *P *= 0.011).

Within this cohort, 367 lymph node-negative patients were considered at low risk of metastasis according to the prevailing guidelines at the time of study accrual and did not receive systemic adjuvant treatment. Of these patients, those that were CTC-positive had a significantly higher risk of metastasis and breast cancer death than those that were CTC-negative (MFS HR = 3.0, *P *= 0.014; BCSS HR = 3.6, *P *= 0.011) (Figure 2). In the subgroup of patients receiving chemotherapy (with or without hormonal treatment; *n *= 155), CTC status was also prognostic (MFS HR = 2.3, *P *= 0.042; BCSS HR = 3.1, *P *= 0.012). For patients treated with hormonal therapy only (*n *= 164), survival differences according to CTC status were not statistically significant (MFS HR = 1.3, *P *= 0.701; BCSS HR = 1.7, *P *= 0.376).

### Circulating tumour cell status versus disseminated tumour cell status

**Figure 3:**
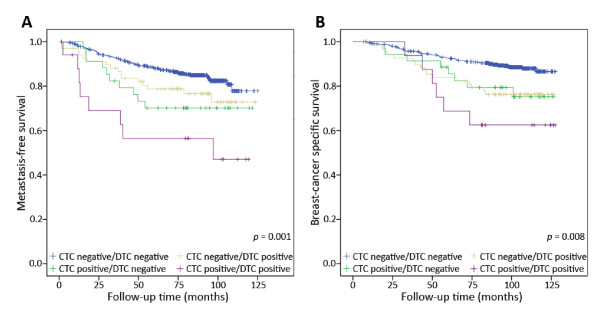
**Kaplan-Meier plots demonstrating the effect of combining circulating and disseminated tumour cell status on metastasis-free survival (**A**) and breast-cancer specific survival (**B**)**. Those patients in whom tumour cells were detected in both the peripheral blood and bone marrow compartments have a significantly poorer survival compared to other groups (MFS = 3.5, *P *= 0.001; BCSS = 3.0, *P *= 0.008).

Previously, we reported the clinical significance of DTC status in this patient cohort (over a shorter follow-up period) by immunocytochemical detection of cytokeratin-positive cells [1]. This allowed the direct comparison of the prognostic value of CTC detection to DTC detection. Of the 733 patients included in the study, the bone marrow samples of 86 patients (11.7%) contained DTCs. In univariate analyses DTC status was also a significant predictor of outcome, with a HR of 1.9 for MFS (*P *= 0.006) and 2.3 (*P *= 0.001) for BCSS (Table 3). However, in the multivariate analyses including CTCs, DTC status fell below the level of significance (Table 4). Patients in whom tumour cells were detected in both peripheral blood and bone marrow had a significantly poorer outcome than those with tumour cells detected in only one or neither compartment (MFS = 3.5, *P *= 0.001; BCSS = 3.0, *P *= 0.008; Figure 3). In addition, those patients who were exclusively CTC-positive or exclusively DTC-positive also had reduced survival as compared to patients who were negative for both (CTC+/DTC- BCSS HR = 2.2, *P *= 0.015; MFS = 1.9, *P *= 0.034. CTC-/DTC+ BCSS HR = 2.1, *P *= 0.003; MFS = 1.5, *P *= 0.127).

A subgroup univariate analysis revealed that DTC status was only prognostic in lymph node-positive patients (MFS HR = 2.1, *P *= 0.004 and BCSS HR = 2.1, *P *= 0.007), and not in lymph node-negative patients (MFS HR = 0.6, *P *= 0.378 and BCSS HR = 1.0, *P *= 0.978). CTC status however, appeared to be prognostic in both groups (MFS HR = 2.1, *P *= 0.015; BCSS HR = 1.9, *P *= 0.045 for lymph-node positive patients, and MFS HR = 2.3, *P *= 0.039; BCSS HR = 2.9, *P *= 0.017 for lymph-node negative patients; Figure 1). When patients were subgrouped by ER, PR, and HER2 status, CTC status, but not DTC status, was significantly associated with survival for ER-negative and/or HER2-negative patients. For ER-positive patients, those with the presence of DTCs experienced reduced BCSS. This was not observed for CTC positive patients. Both CTC and DTC status were associated with MFS (Table 5). Of the 143 ER/PR/HER-negative ("triple negative") patients, CTC status, but not DTC status, was prognostic for both MFS (HR = 2.1, *P *= 0.034 vs HR = 1.1, *P *= 0.869, respectively) and BCSS (HR = 2.4, *P *= 0.019 vs HR = 1.1, *P *= 0.846).

**Table 5:** Prognostic value of CTC and DTC detection in patients subgrouped by ER, PR, and HER2 status (Bold: *P *> 0.05)

			ER		PR		HER2	
			**Positive**	**Negative**	**Positive**	**Negative**	**Positive**	**Negative**

**Metastasis-free survival**		HR	**2.2**	**2.2**	**2.6**	**2.3**	1.2	**2.9**
	CTC status	95% CI	**1.0 to 4.6**	**1.2 to 4.3**	**1.2 to 5.9**	**1.3 to 4.2**	0.5 to 2.9	**1.6 to 5.1**
		*P*-value	**0.037**	**0.015**	**0.018**	**0.006**	0.691	**< 0.001**
		HR	**2.1**	1.1	1.6	1.5	1.2	1.6
	DTC status	95% CI	**1.2 to 3.9**	0.5 to 2.4	0.7 to 3.6	0.8 to 2.9	0.5 to 3.0	0.9 to 2.9
		*P*-value	**0.015**	0.902	0.243	0.179	0.712	0.103
**Breast cancer specific survival**		HR	1.9	**2.4**	2.0	**2.8**	1.4	**2.9**
	CTC status	95% CI	0.7 to 4.8	**1.2 to 4.6**	0.7 to 5.6	**1.5 to 5.2**	0.5 to 3.4	**1.5 to 5.5**
		*P*-value	0.180	**0.01**	0.200	**0.001**	0.504	**0.001**
		HR	**2.9**	1.1	1.4	2.0	1.6	1.7
	DTC status	95% CI	**1.4 to 5.7**	0.5 to 2.5	0.5 to 3.9	1.1 to 3.8	0.7 to 4.0	0.9 to 3.3
		*P*-value	**0.003**	0.832	0.560	0.025	0.268	0.098

CI, confidence interval; CTC, circulating tumour cell; DTC, disseminated tumour cell; ER, estrogen receptor; HER2, human epidermal growth factor receptor 2; HR, hazard ratio; PR, progesterone receptor

## Discussion

We aimed to validate a previously-developed [2,20], highly sensitive multi-marker QPCR-based CTC assay in a large early-stage breast cancer patient cohort with mature outcome data. Bone marrow samples from these patients had previously been assayed for DTCs by immunocytochemical analysis using anti-cytokeratin antibodies [1]. This made it possible to also directly compare the prognostic value of the detection of tumour cells in the peripheral blood versus the bone marrow in the same large patient group.

In this cohort of 733 early-stage breast cancer patients, CTC status was a significant predictor of both metastasis-free and breast cancer-specific survival, in agreement with other studies [2,6,7,26,27]. To our knowledge, this is the largest published study on CTCs with mature outcome data. There was no significant difference in the distribution of many common clinical variables between CTC-positive and CTC-negative patients, barring that CTC-positive patients had tumours which were ER-negative, PR-negative and HER2-positive significantly more often, in agreement with other studies [28]. CTC-positive patients were in addition more likely to have tumour cells present in both their bone marrow, in accordance with the findings of Daskalaki *et al.*, Pierga *et al.*, and Müller *et al. *[7,29,30] (Table 1).

Importantly, multivariate analysis demonstrated that CTC status provided prognostic information independent of other common clinical variables (Table 4). Furthermore, the CTC analysis discriminated outcome among those patients deemed at sufficiently low-risk of distant metastasis by traditional risk assessment to not receive systemic treatment. Indeed, these "low-risk" lymph node-negative CTC-positive patients had a five-year BCSS of 75.0%, versus 93.0% for the CTC-negative patients (Figure 2). The identification of such a poor prognosis subgroup within a group of traditionally-defined "low risk" patients indicates the potential value of CTC detection in routine clinical use in conjunction with other prognostic tests.

Our results show that immunomagnetic tumour cell enrichment followed by multi-marker QPCR analyses on samples of PBMCs stored frozen for up to 15 years provides valuable clinical data. As expected, some cellular and nucleic acid degradation was observed in these samples after their removal from long-term storage, with tumour marker gene expression lower, variance higher (24-fold) and lower frequency of CTC positive cases (7.9%) than that observed with our recent study using fresh material from a similar patient group (*n *= 82) [2]. None of the 25 healthy controls assayed were determined to be CTC-positive, which suggested high specificity. On the other hand, it should be noted that the possibility of occasional tumour marker gene expression in non-malignant cells of breast cancer patients cannot entirely be excluded, which may decrease true specificity in a clinical setting.

Interestingly, while CTC status was correlated with DTC status in these patients, it also provided additional prognostic information and outperformed DTC status in the multivariable analysis (Figure 3, Tables 4 and 5). Keeping in mind the retrospective design of this study, the presented results indicate that the CTC status especially improves the ability to predict outcome in the ER negative subset. It is possible that CTCs and DTCs to some extent reflect distinct aspects of the disease and that their presence and relevance depend on the biology of the underlying breast cancer: bone marrow is a compartment in which tumour cells may remain dormant for many years [17], whereas peripheral blood represents a transitory route for tumours that may be more rapidly proliferating and/or actively migrating. This is consistent with the observation that CTC-positive patients are significantly more likely to have ER-/PR-/HER2+ (Table 1) or triple negative tumours (*P *= 0.003 by Pearson Chi-square), which tend to be more aggressive and exhibit high proliferative capacity [31]. DTCs by comparison, might have higher propensity to remain dormant but viable for extended periods, and, therefore, be more predictive of metastasis or relapse at later time points. This is consistent with our previous study in which the DTC status of a subset of patients from the current cohort was correlated to molecular subtype [32]. It was found that prognostic DTC status was most strongly correlated with patients with the less aggressive Luminal A-type tumours.

In some previous studies CTC status was found to be considerably less prognostic than DTC status [10,11], in contrast to the present results. We cannot exclude the possibility that differences in the prognostic value of DTC detection in different studies may be due to the cytokeratin antibody used [33]. However, the novel CTC detection platform we have employed does offer high sensitivity and specificity by incorporating multi-marker QPCR-based detection and immunomagnetic tumour cell enrichment [20], resulting in increased prognostic power. This strategy also presents one limitation, however; by using anti-EpCAM and anti-ErbB2 Micro Beads for immunoselection, tumour cells that do not express or express low levels of EpCam or HER2 may not be enriched. Indeed, CTCs are known to be biologically heterogeneous (for review see [34]), and able to undergo phenotypic changes during migration. For example, some CTCs may lose EpCam in order to intravasate and reach circulation during epithelial-to-mesenchymal transition (EMT) [35]. Also, the use of an antibody directed against HER2 would favour the selection of HER2 positive CTCs, which may contribute to the observed significant overrepresentation of CTC-positive patients who have HER2-positive tumours.

Although there are biological explanations for differences in the clinical impact of CTCs and DTCs, different methodological principles for the detection of CTCs and DTCs, including the possibility for different sensitivity, might also contribute to incongruent results. Therefore, parallel analysis of CTCs and DTCs with the same methodological principle should be encouraged in future trials.

Our CTC assay was originally developed using fresh PBMCs, and in our previous study demonstrated more than twice the CTC positivity rate in early-stage patients than was observed using the liquid nitrogen-stored material here [2]. The use of our method on fresh PBMCs would, therefore, be expected to further increase the sensitivity and clinical utility of the assay. Future robust prospective studies are warranted to demonstrate this.

## Conclusions

Our results add further evidence for the potential clinical value of CTC and DTC detection in risk stratification in early breast cancer. According to our data, approximately one in five breast cancer patients are likely to be CTC and/or DTC positive at the time of diagnosis and, significantly, a proportion of these will be classed as 'low-risk' by traditional prognostic measures and may not receive any form of systemic treatment, yet would probably derive considerable benefit from it. Our results also suggest that while CTC status may be easier to determine and monitor than DTC status due to the accessibility of the sampling material, both CTC and DTC status provide valuable clinical data that may be indicative of different disease aspects. Together, these results provide evidence that CTC and DTC detection generate prognostic information that would be useful in a clinical setting.

## Abbreviations

BCSS: breast cancer-specific survival; BM: bone marrow; CTC: circulating tumour cell; DTC: disseminated tumour cell; ER: estrogen receptor; HER2: human epidermal growth factor receptor 2; HR: hazard ratio; LDA: Linear Discrimination Analysis; MNC: mononuclear cells; MFS: metastasis-free survival; PBMC: peripheral blood mononuclear cells; PR: progesterone receptor; QDA: quadratic discriminant analysis; (Q)PCR: (quantitative) polymerase chain reaction

## Competing interests

The authors declare that they have no competing interests.

## Authors' contributions

TM and AB carried out CTC assays, performed data analysis and drafted the manuscript. LB, MS, EB and ES carried out DTC assays and the associated analyses. LV and BN were responsible for study design, patient accrual, statistical analysis, and drafting the manuscript. All authors read and approved the final manuscript.
